# Increased therapeutic efficacy of combination of azithromycin and ceftazidime on *Pseudomonas aeruginosa* biofilm in an animal model of ureteral stent infection

**DOI:** 10.1186/s12866-016-0744-1

**Published:** 2016-06-24

**Authors:** Xianfeng Wang, Yongqing Cai, Haiyan Xing, Wei Wu, Guanying Wang, Ling Li, Jianhong Chen

**Affiliations:** Department of Pharmacy, Institute of Surgery Research, Daping Hospital, Third Military Medical University, Chongqing, 400042 China

**Keywords:** Ureteral stent infection, Azithromycin, Ceftazidime, Bacterial biofilm

## Abstract

**Background:**

Infection caused by ureteral stent indwelling is one of the most difficult medical problems, since once bacteria reside in biofilms they are extremely resistant to antibiotics as well as to the host immune defences. In this study we assessed the in vitro and in vivo efficacy of azithromycin and ceftazidime in preventing ureteral stent infection by *Pseudomonas aeruginosa*.

**Results:**

The susceptibility testing with adherent bacteria showed that the biofilm was strongly inhibited by azithromycin treatment, ceftazidime against adherent bacteria in the presence of azithromycin showed the minimum inhibitory concentrations (MICs) and minimum bacteriocidal concentrations (MBCs) dramatically lower than those obtained in the absence of azithromycin. Moreover, ceftazidime plus azithromycin reduced twitching motility and production of rhamnolipid. For the single-treatment groups, in vivo intravenous injection of ceftazidime showed the highest inhibitory effect on bacterial load. Azithromycin prophylactic injection combined with ceftazidime showed increased inhibitory effect on bacterial load than that of each single antibiotic.

**Conclusions:**

Combination of azithromycin and ceftazidime effectively prevent the formation of biofilm and reduced bacteria load of Pseudomonas *aeruginosa* compared to separate treatment of either of these two antibiotics. This combined treatment option have the potential to contribute to the success of *Pseudomonas* biofilm elimination in the clinical environment.

## Background

Ureteral stents are a basic study tool in Urology. They are widely used for treatment of urolithiasis and relief of benign or malignant obstruction [1, 2]. However, when the medical devices was implanted in the urinary tract they can provide conditions for biofilm formation. The longer devices remain indwelling, the greater the probability of bacteria to develop biofilms [1]. Biofilm formation increased the infection risk, morbidity, and encrustation that may result in renal failure and even death [3–5].

*Pseudomonas aeruginosa* is an opportunistic human pathogen frequently linked to infections of indwelling catheters and foreign-body implants [6, 7]. Its biofilm is equiped with an exopolysaccharide matrix that is able to adhere to various biotic and abiotic surfaces [3, 8, 9]. When *P. aeruginosa* have colonized ureteral stent, it is almost impossible to deracinate the biofilm, which increase virulence factors with immune evasion and antibiotic resistance [10, 11]. To eradicate the infection, surgery is necessary to remove the implant but the replacement has a tendency of bacterial recolonization that results in renal insufficiency [6, 10].

Biofilm formation is controlled by a system of bacterial intercommunication, known as quorum sensing (QS). The importance of QS in the pathogenesis of *P. aeruginosa* infection has gained considerable attention in developing antimicrobial strategy [12–14]. When *P. aeruginosa* generate biofilms, the QS-controlled production of tissue-damaging virulence factors such as rhamnolipid can kill incoming polymorphonuclear leucocytes (PMNs), which enhance biofilm formation by release of PMN DNA [15]. The high morbidity and mortality and resistance to conventional antimicrobial drugs in *Pseudomonas*-related biofilm infection needs new urgent therapeutic strategies. The potential clinical value of antibacterial agents that control *P. aeruginosa* infection by impeding QS and adhesion abilities has recently been emphasized. Biofilm models in vitro and in vivo have been utilized to access the susceptibility of *P. aeruginosa* to antimicrobial agents [16–18].

Azithromycin (AZM) has shown potential inhibiting effects on *P. aeruginosa* biofilm with reducing of bacteria virulence factors and adhesion abilities when it was used in sub-inhibitory concentrations in a urinary tract infection (UTI) and cystic fibrosis (CF) model [19, 20]. Ceftazidime (CAZ) also showed QS inhibitory activity, decreasing the production of a range of QS-regulated virulence factors [21]. In the present study, we assessed the in vitro and in vivo potential of AZM and CAZ in preventing ureteral stent biofilm infection induced by a clinical isolate of mucoid *P. aeruginosa*. The experiments were performed using Checkerboard technique to test the in vitro synergistic antimicrobial effects of AZM and CAZ and ureteral stent biofilm infection model to evaluate the in vivo efficacy of combination therapy of AZM and CAZ.

## Results

### Susceptibility testing

The development of adherent biofilm was photometrically corroborated when the bacterial strain showed a mean OD_570 nm_ of 0.786 ± 0.058. CAZ against the adherent bacteria without AZM showed MIC and MBC values of 128.00 and 512.00 mg/L, respectively. CAZ plus AZM showed MICs 16-fold (8.00 mg/L) and MBCs 32-fold (32.00 mg/L) lower (Table 1).

**Table 1:** Antimicrobial activity of CAZ and AZM against adherent cells of clinical isolate of *P. aeruginosa*

Agent	AH165 slime-producing strain (adherent cells)	
	MIC (mg/L)	MBC (mg/L)
AZM	>256	>256
CAZ	128	512
AZM plus CAZ^a^	8	32

^a^Wells pre-treated with AZM (8 mg/L AZM in MH broth for 30 min)

### Synergy studies

In the interaction studies between CAZ and AZM, synergy was confirmed when the range of FIC indexs was 0.192–0.429 in five clinically isolated strains (Table 2).

**Table 2:** Results of combination of CAZ and AZM

*P. aeruginosa* strain	FIC index
AH 165	0.276
AH 374	0.192
AH 108	0.376
AH 626	0.314
AH 431	0.429

The range of drug dilutions used was 0.25–256 mg/L. FIC indexes were interpreted as ≤ 0.5, synergy; 0.5–4.0, indifferent; and >4.0, antagonism

### Effect on twitching motility

**Fig. 1:**
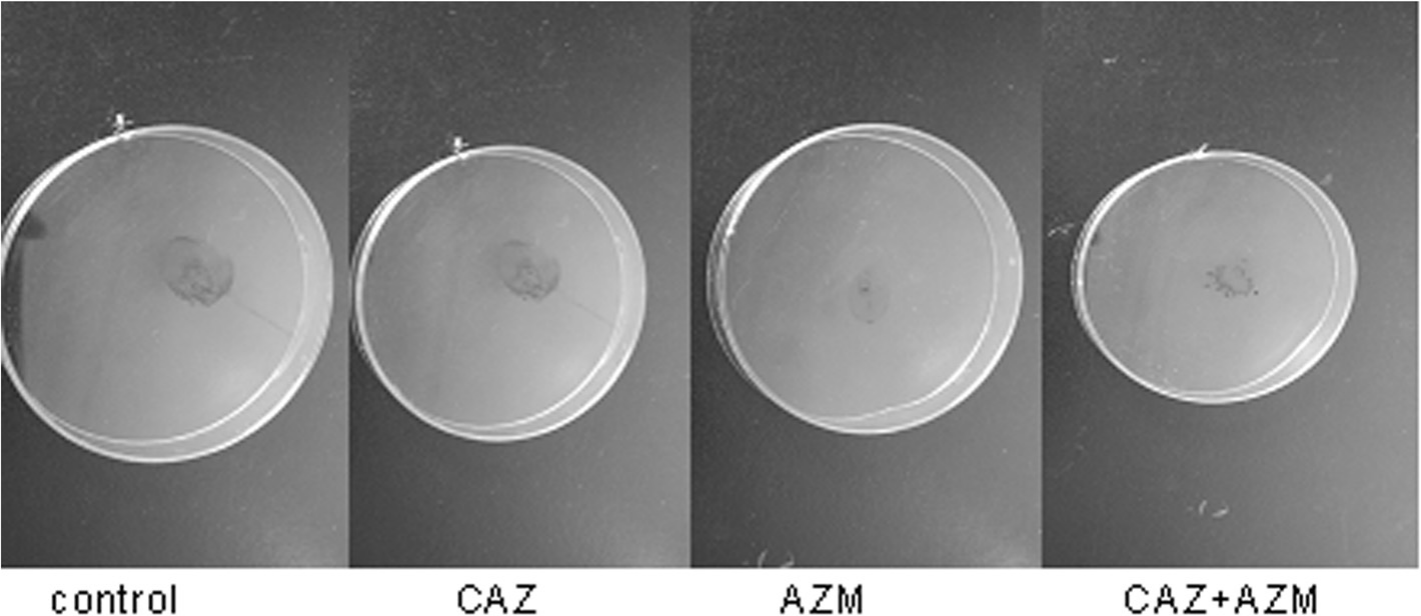
Demonstration of twitching motility of *P. aeruginosa* (AH165). Detailed legend: control- no antibiotic, CAZ- Ceftazidime,AZM- azithromycin

*P.aeruginosa* possesses type IV pili, which enable the organism to recruit adjacent cells through twitching motility and form biofilm [22]. The result indicated that CAZ didn’t reduce twitching motility of *P.aeruginosa*, but combination of CAZ and AZM significantly reduced twitching motility compared to CAZ or AZM (*P* < 0.01, *P* < 0.01) (Table 3, Fig. 1).

**Table 3:** Effect on twitching motility

Drug	Zone diameters(cm)
control	1.16 ± 0.050
CAZ	1.15 ± 0.047
AZM	0.62 ± 0.036^a^
CAZ + AZM	0.34 ± 0.021^a,b,c^

^a^Statistically significant when compared with no antibiotic treated medium(control), *P* < 0.01

^b^Statistically significant when compared with singly CAZ-treated medium, *P* < 0.01

^c^Statistically significant when compared with singly AZM-treated medium, *P* < 0.01

### Inhibition of rhamnolipid production in vitro

**Fig. 2:**
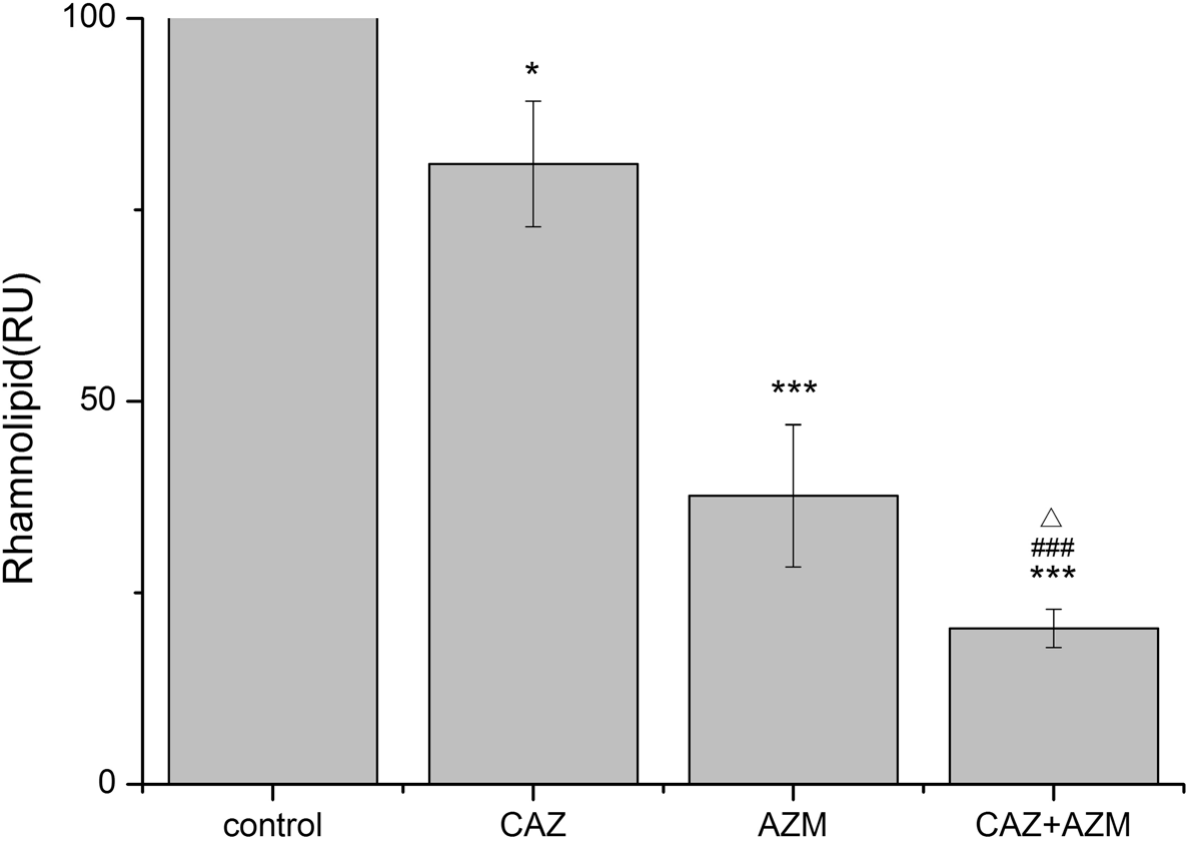
Inhibition of production rhamnolipid of *P. aeruginosa* (AH165). Detailed legend: The concentration of rhamnolipid in the untreated culture was set equal to an index value of 100. The graph is based on the average of the indexes of three independent experiments. *P < 0.05; *** *P* < 0.001 (compared with control); ^###^ *P* < 0.001 (compared with CAZ); ^△^ *P* < 0.05 (compared with AZM)

Rhamnolipid as an important tissue-damaging virulence factor can enhance formation of biofilm and protect the biofilm from host immune defense [15]. The result showed that CAZ or AZM significant inhibited production of rhamnolipid compared to no antibiotic (*P* < 0.05, *P* < 0.01). Moreover, combination of CAZ and AZM significantly reduced production of rhamnolipid compared to CAZ or AZM (*P* < 0.01, *P* < 0.05) (Fig. 2).

### In vivo studies

**Fig. 3:**
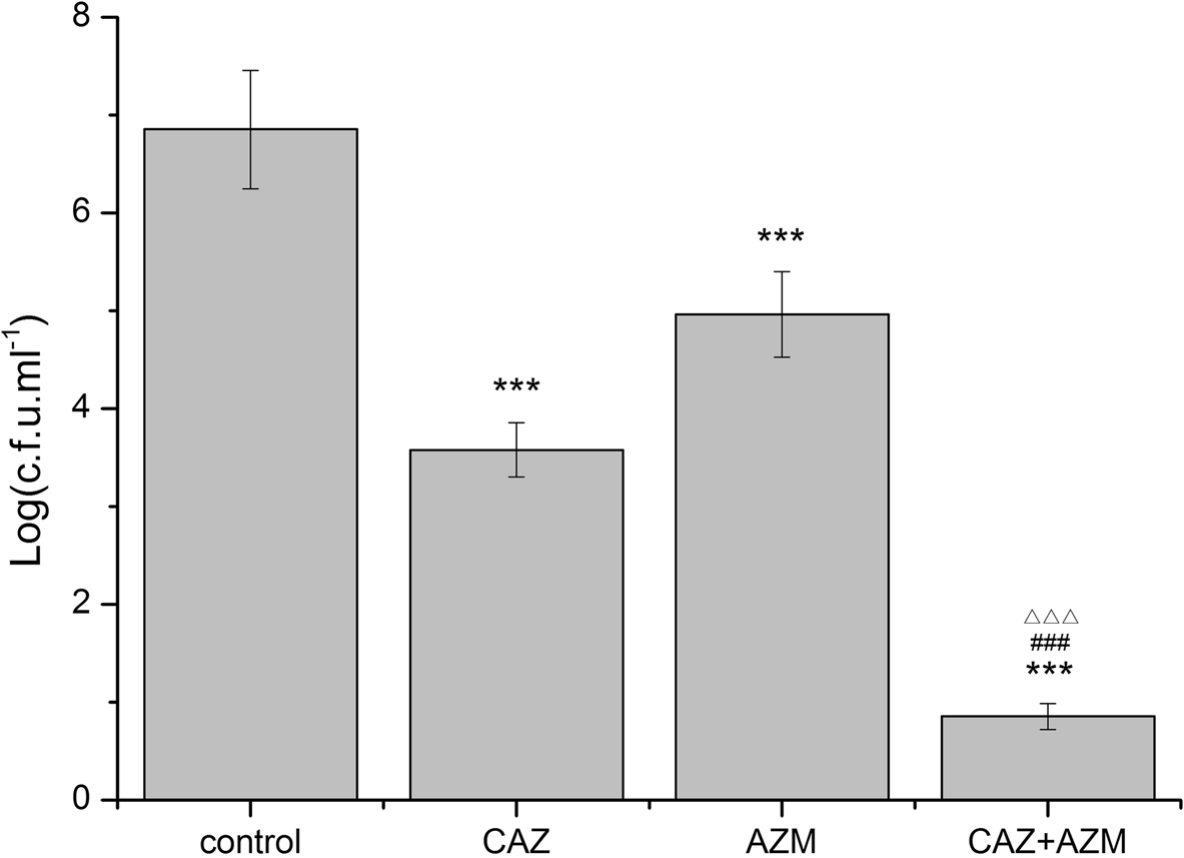
Activity of CAZ and AZM against *P. aeruginosa* (AH165) clinical isolate in a rat model of ureteral stent infection (stent cutrue). Detailed legend: Ureteral stents were explanted at day 5 following implantation. Bacterial counts are given in terms of log_10_ cfu/mL. The limit of detection for the method was ≤1 cfu/mL. *** *P* < 0.001 (compared with control); ^###^ *P* < 0.001 (compared with CAZ); ^△△△^ *P* < 0.05 (compared with AZM)

**Fig. 4:**
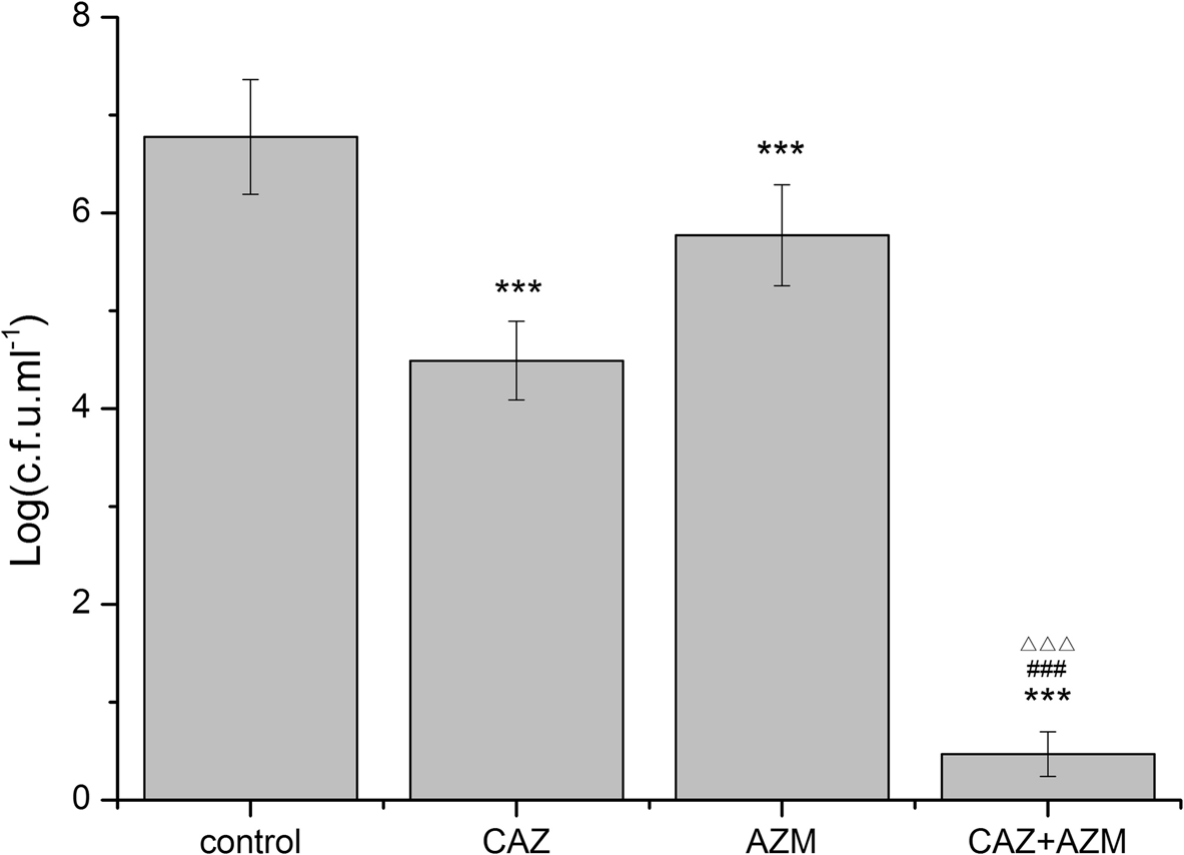
Activity of CAZ and AZM against *P. aeruginosa* (AH165) clinical isolate in a rat model of ureteral stent infection (urine cutrue). Detailed legend: The urine cultures were performed 24 h after ureter stent placement using a transvesical sample taken by an insulin syringe. Bacterial counts are given in terms of log_10_ cfu/mL. The limit of detection for the method was ≤10 cfu/mL. *** *P* < 0.001 (compared with control); ^###^ *P* < 0.001 (compared with CAZ); ^△△△^ *P* < 0.05 (compared with AZM)

The findings that synergistic activity of AZM and CAZ was noted in in vitro studies encouraged us to conduct combination therapy in an animal model of *P. aeruginosa* stent biofilm infection. The result showed that none of the animals suffered from stent infection in the control group (C_0_) without bacterial challenge. In contrast, all rats suffered from graft infection in challenged control group (C_1_), with quantifying value showing 6.85 ± 0.602 log_10_ cfu/mL. Rats that treated with CAZ showed stent bacterial counts of 3.58 ± 0.274 log_10_ cfu/mL. Rats that prophylactically treated with AZM showed stent bacterial counts of 4.96 ± 0.438 log_10_ cfu/mL. Interestingly, CAZ combined with AZM showed efficacies higher than that of each single drug, without a presence of bacterial counts (*P* < 0.001) (Fig. 3). Similarly, urine cultures were negative in both control group (C_0_) and combined therapy group. Urine cultures results also showed that a significant reduction in log count of 4.49 ± 0.403 and 5.77 ± 0.518 in the groups of rats treated intravenously respectively with CAZ and AZM, as compared with 6.78 ± 0.586 in the challenged control group (C_1_)(*P* < 0.001) (Fig. 4). Drug related adverse effects and dead rats were not observed in any group throughout the study.

## Discussion

Infection caused by ureteral stent indwelling is one of the most difficult medical problems, since once bacteria reside in biofilms they are up to 1000-fold more tolerant to antibiotics. Moreover, ureteral stent infection can not be easily diagnosed. It is reported that 90 % indwelling silicone double J stents were occupied by adherent bacteria; however, only 27 % of urinary infection was clinically detected [23]. It is also reported that only 30 % of stent indwelling patients were found to have bacteriuria, but 68 % were found stent colonization [24]. An additional problem in ureteral stent biofilm associated infections is the development of encrustation and consecutive obstruction [6, 7]. Biofilm that contribute to antibiotic resistance has a variety of attributes, such as, a mixed population of rapidly and slow- or non-growing bacteria, an exopolysaccharide matrix, a change in gene expression [25]. Biofilm of bacterial colonization also acts as a physical barrier against host defense [26, 27]. As biofilms are increasingly resistant to antibiotics, making monotherapy ineffective, combination therapy is essential for eradication of infection [28].

The aim of the present study was to assess the efficacy of AZM and CAZ (an anti-*Pseudomonas* agent) in preventing ureteral stent-associated *Pseudomonas* infection. We hypothesize that AZM might inhibit the virulence factors of the bacteria by preventing initial adherence to the implants or biofilm formation thereby enhance the activity of CAZ. Our data showed that both AZM and CAZ significantly reduced bacterial load on ureteral stent tissue. When AZM was combined with CAZ, no evidence of *Pseudomonas* was detected on the stent or in the urine. In vitro studies also demonstrated that AZM enhanced the effect of CAZ against some *Pseudomonas* strains, in accordance with reported results. Moreover, CAZ plus AZM reduced twitching motility and production of rhamnolipid.

In fact, although AZM has no antimicrobial activity against *P. aeruginosa* at therapeutic concentrations, our study showed that AZM may exert activity against this microorganisms. It can exert its effect on *P. aeruginosa* biofilm formation by reducing bacterial virulence factors at sub-inhibitory concentrations and influencing the flux of 3-oxo-C_12_-HSL through changed membrane permeability [29]. It has also been reported that AZM is able to reduce IV pili-confered twitching motility which contributes to bacterial adherence and generation of biofilm [20]. CAZ is a third generation cephalosporin effective against Gram-negative bacteria especially *P. aeruginosa* and also a QS-inhibitor [21, 30], but it is insensitive to *P. aeruginosa* in adherent form [28]. In the combination, AZM has augmented the activity of CAZ in vitro [28], but the in vivo efficacy has never been validated. We exteneded the study to an in vivo *P. aeruginosa* ureteral stent biofilm model. The doseage levels and the delivery method of CAZ and AZM used in this study are based on reported studies methods which mimic clinical practice [20, 31]. Results suggest that AZM was effective in preventing biofilm formation, and combined with CAZ, it was able to improve CAZ activity through reduction of bacterial adherence and virulence factor production. Therefore, our investigation indicates that injection intravenous of AZM prophylactically is able to prevent biofilm formation through reducing bacterial adherence and virulence factor production. Subsequect intravenous injection of CAZ after implantation aids to the clearance of *P. aeruginosa*.

## Conclusions

In conclusion, prevention of *P. aeruginosa* bioflim formation and reduction of bacteria load of *Pseudomonas aeruginosa* was enhanced by CAZ combined with AZM compared to separate treatment of either of these two antibiotics. Combination therapy of azithromycin and ceftazidime have the potential to contribute to the success of *Pseudomonas* biofilm elimination in the clinical environment.

## Methods

### Bacterial strains

Five clinical isolates of *P. aeruginosa* were collected from urine samples of patients having catheter-associated UTI attending the Institute of Surgery Research, Daping Hospital, Third Military Medical University, Chongqing, China. This study protocol was approved by the Ethics Committee in Research of Institute of Surgery Research, Daping Hospital, Third Military Medical University. A mucoid strain of *P. aeruginosa* (AH165) was used in this study, another four strains were used for interaction studies. All strains were stocked in 10 % glycerol at −80 °C.

### Antimicrobial agents

Sterile stock solution of azithromycin (AZM) (Sigma-Aldrich, USA) and ceftazidime (CAZ) (Sigma-Aldrich, USA) was prepared according to the manufactures’ instructions and stored at −80 °C. Working solutions were prepared in Mueller-Hinton broth at 512 mg/L for CAZ,8192 mg/L for AZM. These working solutions were serial 2-fold diluted with Mueller-Hinton broth and distributed in a 96-well microtiter plate. The antibiotic dosages of CAZ and AZM used in the in vivo experiments were 100 mg/kg and 350 mg/kg, respectively [20, 31].

### Susceptibility testing with planktonic bacteria

MICs and MBCs were determined by broth microdilution using the 2-fold dilution method according to CLSI guidelines [32]. The antibiotic concentrations tested ranged from 0.5 to 256 mg/L for CAZ and from 2 to 4096 mg/L for AZM.

### Biofilm-forming capacity

Biofilm-forming capacity was determined as previously described [22]. Prior to testing, the strains were subcultured in tryptic soy broth (TSB) (Sigma Fluka, USA) and incubated overnight after retrieval from −80 °C. A bacterial suspension in TSB was prepared with an inoculum density equivalent to 10^6^ cfu/mL. Afterwards, 50 μL of TSB were inoculated into each well of polystyrene 96-well microtiter plate (Corning, USA) containing 150 μL of TSB/2 % glucose. The growth medium was discarded after 24 h of incubation at 37 °C, and then each well was washed three times with sterile PBS to remove free cells. The remaining attached bacteria were fixed for 15 min with 200 μL of 99 % methanol per well, and the wells were emptied and left to dry. Wells were then stained with 0.2 mL of Crystal Violet (2 %, w/v) for 5 min at room temperature. Excess dye was removed by washing the well with running tap water. The plates were air dried and the dye taken up by the biofilm cells was extracted with 0.2 mL of glacial acetic acid (33 %, v/v) per well. Absorbance was measured at 570 nm by using Multiskan Spectrum (Thermo Scientific, Finland). The same experiment was performed three times with and without the addition of 8 mg of AZM in Mueller-Hinton (MH) broth in each well. Biofilm capacity was calculated as three standard deviations above the mean OD of the negative control.

### Susceptibility testing with adherent bacteria

The MIC and MBC were determined with modifications for use in the biofilm test. Biofilms were washed with sterile PBS in order to remove non-binding cells. Subsequently, 200 μL of MH broth containing serial 2-fold dilutions of antibiotic were added to each well of tissue-culture-treated polystyrene 96-well microtiter plate containing adherent organisms. The plates were incubated for 18 h at 37 °C in air. The MIC was taken as the lowest CAZ concentration at which observable growth was inhibited. To determine the MBC, the MH broth containing CAZ was removed from each well and replaced with antibiotic-free MH broth; the plates were incubated again for 18 h at 37 °C in air. The MBC was taken as the lowest concentration of AZM that resulted in no bacterial growth following removal of the drug. In addition, to investigated the effect of AZM pre-treatment on bacterial susceptibility to CAZ, the MIC and MBC of CAZ were again determined after pre-treatment of cells by incubation for 30 min at room temperature in 8 mg/L AZM solution immediately before susceptibility testing.

### Twitching motility assay

Twitching motility was assayed on freshly prepared Luria-Bertani agar plates (1 % Bacto agar) containing medium with 4 mg/L CAZ, 8 mg/L AZM, 4 mg/L CAZ plus 8 mg/L AZM, or no antibiotic (control) [20]. For the motility assay, organisms were grown overnight and stabbed with a sterile toothpick through the agar layer to the bottom of the Petri dish. The plates were incubated at 37 °C for 48 h and washed gently with tap water to remove any unattached cells. Then the attached cells were stained with crystal violet (0.1 %,W/V). The diameter of the stained zone was measured to assess the twiching motility.

### Quantification of rhamnolipid

Rhamnolipid B was measured with an Agilent 1100 series high-pressure liquid chromatography connected to a Micromass LCT time-of-flight mass spectrometer. The total ionization current was determined on the [M + NH_4_]^+^ ion at 668.4 over the 7 s over which rhamnolipid B was eluted. Before determination of rhamnolipid B, *P. aeruginosa* (AH165) cultures were grown to an OD_600_ of 2.0 with 4 mg/L CAZ, 8 mg/L AZM, 4 mg/L CAZ plus 8 mg/L AZM, or no antibiotic (control). Cells were harvested by centrifugation, and the supernatants were filter sterilized (TPP syringe filter; pore size, 0.22 μm). The concentration of rhamnolipid in the untreated culture was set equal to an index value of 100.

### Synergy studies

To test the antibiotic combinations of AZM and CAZ, five clinical isolate strains were used in the studies. Checkerboard arrangements of AZM and CAZ were prepared in 96-well polypropylene microtitre plates. In the Checkerboard technique, two drugs are compared in microtitre wells the drug concentrations equal to, above, and below the MIC of the drugs being tested. The fractional inhibitory concentration (FIC) index for combinations of two antimicrobials was calculated as follows [33, 34]:

$$ \mathrm{F}\mathrm{I}\mathrm{C}\ \mathrm{of}\ \mathrm{C}\mathrm{A}\mathrm{Z}\ \left(\mathrm{F}\mathrm{I}{\mathrm{C}}_{\mathrm{A}}\right) = \mathrm{MIC}\ \mathrm{of}\ \mathrm{C}\mathrm{A}\mathrm{Z}\ \mathrm{in}\ \mathrm{combination}/\mathrm{MIC}\ \mathrm{of}\ \mathrm{C}\mathrm{A}\mathrm{Z}\ \mathrm{alone}; $$

$$ \mathrm{F}\mathrm{I}\mathrm{C}\ \mathrm{of}\ \mathrm{A}\mathrm{Z}\mathrm{M}\left(\mathrm{F}\mathrm{I}{\mathrm{C}}_{\mathrm{B}}\right) = \mathrm{M}\mathrm{I}\mathrm{C}\ \mathrm{of}\ \mathrm{A}\mathrm{Z}\mathrm{M}\ \mathrm{in}\ \mathrm{combination}/\mathrm{MIC}\ \mathrm{of}\ \mathrm{A}\mathrm{Z}\mathrm{M}\ \mathrm{alone}. $$

The sum of fractional inhibitory concentration (FICs) indices of two compounds in the combination was calculated as follows:

$$ \mathrm{F}\mathrm{I}{\mathrm{C}}_{\mathrm{A}} + \mathrm{F}\mathrm{I}{\mathrm{C}}_{\mathrm{B}} = \mathrm{F}\mathrm{I}\mathrm{Cs} $$

Synergism = FICs ≤ 0.5; antagonist = FICs ≥ 4; additive = FICs > 0.5 and ≤ 1; indifference = 1 < FICs < 4.

### Animals

Adult female Wistar rats (weight range 190–240 g) were provided by Center for Experimental Animals of Third Military Medical University (Chongqing, China) and were maintained on standard rat chow and water *ad libitum* before the challenge. The animal studies were carried out in accordance with the guidelines for the Ethical Treatment of Laboratory Animals. All procedures of rat care and handling were in accordance with accepted standard operating procedures of the Third Military Medical University. Animals study was approved by the Animal Care and Utilization Committee of the Third Military Medical University.

### Ureteral stent infecion model

Rats were randomly divided into five groups including a control group (C_0_) without bacterial challenge to evaluate the sterility of the surgical procedure, a challenged control group (C_1_) that did not receive any antibiotic prophylaxis, and three challenged groups that received intravenous CAZ 100 mg/kg immediately after stent implantation; intravenous AZM 350 mg/kg prophylactically before implantation; and intravenous CAZ plus intravenous AZM at the above concentrations. Each group contained ten rats.

Ureteral stent implants were prepared as described previously by Daniele Minardi et al. [35] with some modifications. Rats were anaesthetized by an intramuscular injection of ketamine and xylazine (30 mg/kg and 8 mg/kg, respectively), then sterile ureteral stent implants with a size of 0.2 cm^2^ (Porges-Mentor, France) were used and inserted into the bladder. Before stent implantation, some of rats were injected intravenously with 350 mg/kg AZM once every 24 h from day 2 pre-insertion to day 5 post-insertion; After the surgical intervention, a saline solution (1 mL) containing 2 × 10^7^ cfu/mL of *Pseudomonas* strain was inoculated into the bladder using a tuberculin syringe. After stent implantation, some of the animals received intravenous CAZ immediately once every 24 h until to day 5 post-insertion. On the basis of previous experiments demonstrating peak bacterial growth and biofilm formation within 72 h [36], all ureteral stents were explanted at day 5 following implantation. Toxicity was evaluated on the basis of the presence of any drug related adverse effects, i.e. anorexia, weight loss, fever, vomiting, diarrhea, behavioral alterations and local signs of local inflammation.

### Assessment of the infection

To verify sterility or infection of rats, urine cultures were performed through a transvesical sample taken by an insulin syringe 24 h after ureteral stent placement. The explanted ureteral stents were washed in sterile saline solution and sonicated for 2 min in phosphate-buffered saline solution to remove the adherent bacteria from the grafts. After the ultrasound treatment, 100 μL of the PBS-bacteria solution was serial diluted and plated on McConkey agar plates. The plates were incubated at 37 °C for 48 h before determination of cfu per implant. The limit of detection for this method was approximately 10 cfu/mL.

### Statistical analysis

Values of MIC and MBC are presented as the geometric mean of three separate experiments. To compare the bacteria counts (log_10_ cfu) between groups of rats in in vivo experiments, quantitative culture results were presented as mean ± S.D and Tukey–Kramer Honestly Significant Difference Test was used for calculating *P* value. Significance was accepted when the *P* value was 0.05.

## Abbreviations

AZM, azithromycin; CAZ, ceftazidime; CF, cystic fibrosis; FIC, fractional inhibitory concentration; MBCs, minimum bacteriocidal concentrations; MH, Mueller-Hinton; MICs, minimum inhibitory concentrations; *P. aeruginosa*, *Pseudomonas aeruginosa*; PMNs, polymorphonuclear leucocytes; QS, quorum sensing; TSB, tryptic soy broth; UTI, urinary tract infection
